# Green synthesis of CuO nanoparticles using mangrove leaves for targeted delivery of platinum (II) therapeutics

**DOI:** 10.1038/s41598-026-67347-7

**Published:** 2026-09-08

**Authors:** Shahdan Abdelkareem, Aly Reda, Mayyada M. H. El-Sayed, Tamer Shoeib

**Affiliations:** Department of Chemistry, The American University in Cairo, New Cairo, 11835 Egypt

**Keywords:** Green synthesis, Plant-mediated synthesis, *Avicennia marina* (Mangrove), Drug delivery, Platinum chemotherapeutics, Biotechnology, Cancer, Chemistry, Materials science, Nanoscience and technology

## Abstract

Copper oxide nanoparticles (CuO NPs) synthesized via green chemistry using *Avicennia marina* mangrove leaf extract represent an environmentally sustainable approach to generating biocompatible nanomaterials for pharmaceutical applications. This study utilized the phytochemical-rich extract as both a reducing and capping agent to produce quasi-spherical monoclinic CuO NPs with a diameter range of 35–87 nm. The resulting nanoparticles possessed a specific surface area of 4.5 m² g⁻¹ with a mesoporous structure (pore volume of 0.018 cm³ g⁻¹), making them ideal candidates for drug loading applications. The CuO NPs were subsequently evaluated as nanocarriers for two clinically relevant platinum (II) chemotherapeutics: cisplatin and oxaliplatin. Characterization via Fourier-transform infrared spectroscopy confirmed successful coordination of platinum drug ligands to surface Cu/O sites, while Brunauer–Emmett–Teller analysis demonstrated a substantial decrease in surface area upon drug loading, indicating effective pore occupancy. Cisplatin demonstrated an entrapment efficiency of 28% with a loading capacity of 56 mg/g, while oxaliplatin exhibited comparable loading performance. The in vitro release profiles of cisplatin and oxaliplatin from mangrove-derived green CuO NPs show sustained release, with cisplatin reaching 70% cumulative release within 20 h and oxaliplatin approaching 80% in the same period. The release kinetics follow first-order behavior for cisplatin and Higuchi model for oxaliplatin, indicating that release is predominantly governed by diffusion from the mesoporous CuO matrix. These findings establish mangrove-derived CuO nanoparticles as a promising carrier for sustainable platinum-based drug delivery, offering an environmentally friendly approach and enhanced therapeutic potential while eliminating the need for toxic precursors and energy-intensive conventional synthesis methods.

**Supplementary Information:**

The online version contains supplementary material available at https://doi.org/10.1038/s41598-026-67347-7.

## Introduction

Green nanotechnology leverages renewable biological resources to create functional nanomaterials while minimizing environmental impact. Among metal oxides, copper-oxide nanoparticles (CuO NPs) stand out for their intrinsic antimicrobial and anticancer activities, high surface reactivity, and cost-effectiveness^1^. Conventional CuO synthesis routes, however, depend on toxic reagents, high-temperature calcination, and generate significant waste. Plant-mediated “green” synthesis circumvents these drawbacks by exploiting naturally occurring phytochemicals as dual reducing/stabilizing agents, affording biocompatible nanoparticles under benign conditions^2^. Green-synthesized nanoparticles offer several critical advantages over their conventionally produced counterparts: (i) the elimination of toxic reducing agents reduces environmental and cytotoxic risk; (ii) moderate synthesis temperatures contrast with the high-temperature calcination or hydrothermal conditions required for chemical routes, substantially lowering energy consumption; (iii) plant extracts are renewable, low-cost, and available at scale, supporting sustainable manufacturing^2,3^. Plant-mediated synthesis of CuO NPs has advanced significantly over the last decade, with researchers demonstrating reproducible control over particle morphology, crystallinity, and surface chemistry by tuning extract concentration, temperature, and precursor ratios, resulting in nanoparticles with improved bioactivity and colloidal stability^2,4,5^.

Recent studies validate the anticancer potential of green-synthesized CuO nanoparticles but remain limited in scope and mechanistic depth: plant-mediated CuO reports demonstrate cytotoxicity against various cancer lines (e.g., Rhazya stricta and Aloe vera extracts^6,7^ yet primarily perform single drug cytotoxic screens without drug-loading or comparative analyses^8,.^ Other works have begun loading therapeutics (microwave-assisted Pt@CuO^10^ curcumin@CuO–halloysite^11^ but rely on hybrid supports or non-fully plant-mediated routes and focus on single agents or single synthetic platforms, limiting direct clinical translatability and systematic comparison across clinically used Pt(II) drugs. Therefore, a clear gap exists for a fully plant-mediated CuO nanoplatform that: (i) compares multiple clinically approved platinum(II) drugs on the same carrier, (ii) provides rigorous surface characterization and kinetic investigation of drug–nanoparticle interactions, and (iii) quantifies loading/release mechanisms to support translational relevance. This study addresses this gap by evaluating the interaction of *Avicennia marina*–derived CuO NPs with cisplatin and oxaliplatin using comprehensive physicochemical and kinetic analyses.

Mangrove species flourish in salty, oxidative conditions and accumulate a variety of secondary metabolites, including polyphenols, flavonoids, and terpenoids, which are particularly efficient in nanoparticle nucleation and cappin^4,12^. In this study, we used *Avicennia marina*, a salt tolerant pioneer mangrove species widely distributed along tropical and subtropical coastlines including the Red Sea, that thrives in saline, oxidative intertidal zones and is readily available from coastal ecosystems like Ras Mohammed National Park^13^. Its leaves are rich in hydroxyl- and carbonyl-containing secondary metabolites, particularly polyphenols, flavonoids, and terpenoids^13,14^*. Avicennia marina* leaf extract contains numerous hydroxyl- and carbonyl-rich chemicals that chelate Cu²⁺ ions, donate electrons for reduction, and stabilize the CuO surface^15,16^. Specifically, the phenolic hydroxyl groups of polyphenols and flavonoids act as primary electron donors during Cu²⁺ reduction, undergoing oxidation to their corresponding quinone forms while chelating copper ions and templating nanoparticle growth. The resulting quinone-rich and carbonyl-bearing organic layer remains adsorbed on the CuO surface as a stabilizing capping shell, as confirmed by FTIR. This phytochemical shell improves colloidal stability and supports drug distribution by providing hydrogen-bond donors and acceptors that supplement direct Pt-Cu interaction in maintaining platinum drug molecules^17–19^*. Avicennia marina* distinguishes itself from other mangrove species used in nanomaterial synthesis, such as *Bruguiera gymnorrhiza* and *Rhizophora mucronata*, by having an exceptionally high polyphenol and flavonoid content under saline stress, which improves its reducing capacity and provides a denser organic capping layer, a property that is especially useful for drug retention applications^16,19^.

Platinum-based drugs remain a cornerstone of solid-tumor treatment, although their clinical efficacy is limited by fast hydrolysis, systemic toxicity, and nonspecific distribution.^20^ Nanocarrier techniques aim to increase the therapeutic index by increasing drug stability, permitting controlled release, and improving tumor targeting^10^. A range of nanocarrier platforms have been evaluated for platinum drug delivery. Liposomal cisplatin formulations (e.g., Lipoplatin, SPI-77, Aroplatin) can reduce nephrotoxicity compared to free drug and have been tested in clinical trials^20–.^ However, their clinical development has been limited by factors such as insufficient drug release at the tumor site^20,23^. Meanwhile, biodegradable polymers such as PLGA and chitosan are being extensively researched for drug delivery, as they provide customizable release patterns and adaptability for a variety of medicinal agents. Encapsulating highly hydrophilic drugs, such as cisplatin, into ordinary PLGA or chitosan nanoparticles is challenging, often resulting in low drug loading and encapsulation efficiencies^24,25^. CuO NPs are appealing in this setting because: (1) surface oxygen atoms offer coordination sites for Pt(II) complexes, promoting robust adsorption^26^. (2) mesoporosity allows for large drug loading; and (3) the particles’ inherent ROS-mediated cytotoxicity may synergize with platinum treatment^27,28^.

Despite these advances, significant and specific gaps remain unaddressed in literature. First, while *Avicennia marina* has been used to synthesize silver, gold, and zinc oxide nanoparticles^29,30^, and the species has demonstrated direct anticancer potential^31^, no study has systematically evaluated *Avicennia marina*-derived CuO NPs as nanocarriers for clinically approved platinum(II) chemotherapeutics. Second, the overwhelming majority of green-synthesized nanocarrier studies assess only a single drug on a given platform, precluding any understanding of how molecular structure governs drug–nanoparticle affinity. The absence of comparative multi-drug evaluation represents a critical knowledge gap, since cisplatin and oxaliplatin, despite sharing the Pt(II) center, differ markedly in their ligand geometry, polarity, and molecular footprint, properties that are expected to yield measurably different loading efficiencies and release profiles. Third, the mechanistic role of the phytochemical organic capping layer deposited by *Avicennia marina* extract in modulating drug–surface interactions, adsorption energetics, and release kinetics has not been systematically probed. Understanding whether the capping layer promotes or retards drug binding is essential for rational design of plant-mediated nanocarriers. The present study directly addresses all three of these gaps.

This work addresses these gaps through four integrated objectives: (i) the synthesis of CuO NPs via a fully plant-mediated green route using *Avicennia marina* leaf extract, avoiding toxic precursors or energy-intensive calcination steps; (ii) comprehensive physicochemical characterization of the resulting nanoparticles, including XRD, SEM/EDX, FTIR, BET/BJH, and zeta potential, before and after platinum drug loading, to establish a complete structural baseline; (iii) a direct comparative evaluation of cisplatin and oxaliplatin loading efficiency, surface interaction mechanisms, and in vitro release kinetics on the same nanoplatform, quantified through UV–Vis spectrophotometry and kinetic modeling; and (iv) characterization of drug–nanoparticle interactions via FTIR and BET, with observations of potential hydrogen‑bonding contributions from the residual capping layer. The novelty of this study lies in these aspects: it is the first report combining the specific phytochemical profile of *Avicennia marina* with CuO’s inherent anticancer properties for platinum drug delivery; it provides the first systematic comparison of cisplatin versus oxaliplatin loading on a green CuO platform, revealing their differential binding. The study establishes mangrove-derived CuO NPs as a promising, sustainable, and clinically translatable platform for platinum-based chemotherapy.

## Experimental section

### Materials

The leaves of fresh mangrove (*Avicennia marina* (Forssk.) Vierh) were collected from Ras Mohammed National Park on the southern shore of Sinai (Aqaba Gulf), 27.73° N, 34.25° E, with no required permissions for collection. Formal identification of the mangrove used in this study was undertaken by Dr. Mohamed Massed Hejazi at the Department of Marine Science, Suez Canal University, Ismailia, Egypt. Voucher samples have also been deposited at the Botany Herbarium Laboratory of the same department. Plant collection and handling were conducted in accordance with the International Union for Conservation of Nature Policy Statement on Research Involving Species at Risk of Extinction and the Convention on International Trade in Endangered Species of Wild Fauna and Flora.

Copper (II) sulfate pentahydrate (CuSO₄·5 H₂O, ≥ 99%) were purchased from Fisher Scientific, UK and used as received, cisplatin (cis-[PtCl₂(NH₃)₂], 99%), and oxaliplatin (trans-[(1R,2R)-cyclohexane-1,2-diamine]oxalatoplatinum(II), 99%), were sourced from Shandong Boyuan Pharmaceutical Co., Ltd (Shandong, China).

### Methods

#### Preparation of mangrove leaf extract

After being moved into damp plastic bags, the fresh mangrove leaves were washed then were allowed to dry air, then were crushed in an electric mortar. The resulting powdered samples were stored at -20 °C for later use. To obtain a uniform particle-size fraction, 12 g of ground *Avicennia. marina* leaves were sieved through 100 mesh and subsequently suspended in 250 mL of distilled water. The suspension was heated for 30 min at 60 °C and subsequently filtered.^17^.

#### Green synthesis of CuO NPs

**Fig. 1:**
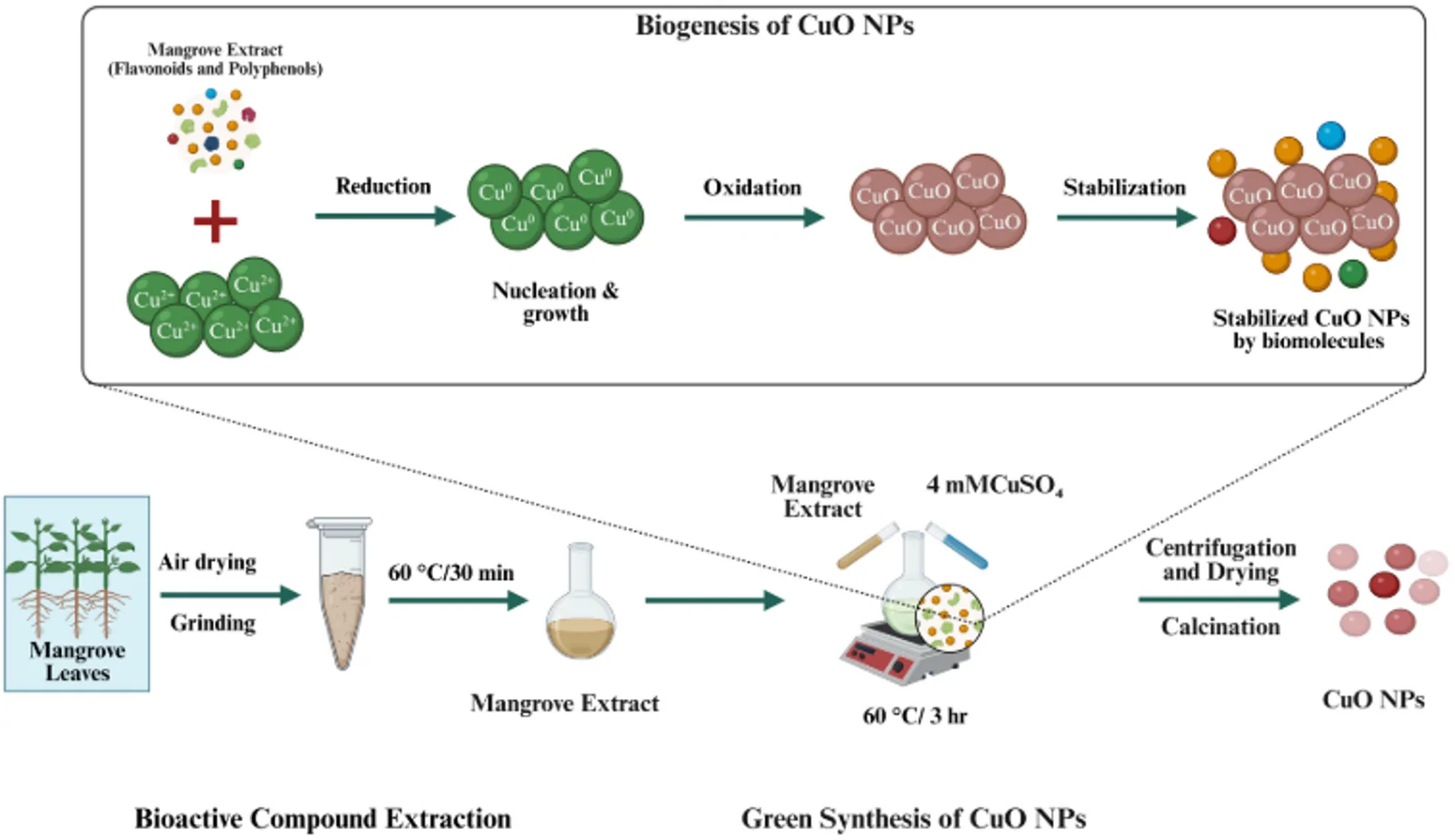
Preparation scheme for the green synthesized CuO NPs.

In a 250 mL Erlenmeyer flask, 30 mL of the prepared *Avicennia marina* leaf extract was added to 100 mL of a 4 mM aqueous solution of copper sulfate pentahydrate (CuSO₄·5 H₂O). The reaction mixture was maintained at 60 °C with constant stirring for 3 h.^17^ The color change from blue to green indicated the formation of copper oxide nanoparticles.^32^ After completion of the reaction, the mixture was allowed to cool down to room temperature. The nanoparticle suspension was centrifuged at 7000 rpm for 20 min at 4 °C (Hermle Z036-HK, Germany). The supernatant was discarded, and the precipitate was washed three times with distilled water. The washed nanoparticles were dried in an oven (Heratherm OGS100, Therma Scientific, USA) at 100 °C for 6 h to remove moisture. The dried nanoparticles were annealed in a muffle furnace (Neycraft Vulcan 3-550 burnout furnace, USA) at 600 °C for 2 h. The phytochemicals present in the mangrove extract, particularly polyphenols and flavonoids, may facilitate the bioreduction of Cu(II) ions by donating electrons through their phenolic hydroxyl groups. These biomolecules have been reported to function as both reducing and capping agents in plant-mediated Cu/CuO nanoparticle synthesis. The copper species formed in the process can then be oxidized to CuO in the reaction medium and/or during thermal treatment, while the oxidized phytochemicals remain adsorbed on the nanoparticle surface, thereby stabilizing the particles and limiting agglomeration.^9,15,33^ A schematic mechanism summarizing the reduction, nucleation and growth, oxidation, and biomolecule-mediated stabilization steps of CuO NP formation by the mangrove extract is presented in Fig. 1. The resulting CuO nanoparticles were stored for subsequent characterization and further use.

#### Drug loading onto CuO NPs

Masses of 35 mg of oxaliplatin and 12 mg of cisplatin were each individually dissolved in 5 mL of deionized water with mild stirring until completely dissolved, creating an aqueous stock solution of each. CuO NPs were added to the saturated drug solution of each at a 2:1 nanoparticle-to-drug ratio (m/m). The mixture was then stirred at 300 rpm for 48 h in the dark. The solutions were then centrifuged at 4000 rpm for 30 min, and the supernatant was utilized for quantitative analysis using UV-visible spectroscopy. The same procedure was repeated, and the solutions of CuO NPs and the drugs were left for 16 h with stirring at 300 rpm and 35 °C until complete evaporation. The drug-loaded powder was kept at 4 °C ^24^then utilized to perform several characterizations.

#### Physicochemical properties of* Avicennia marina* leaves

The aqueous extract of *Avicennia marina* leaves was characterized for its physicochemical properties and elemental composition. The nitrogen analyzer revealed nitrogen content of 1.37% and crude protein content of 7.95%, determined using LECO FP-528 elemental analyzer. Moisture content was determined to be 10.57% using Mettler Toledo moisture balance, which falls within the acceptable range for dried plant material^34^ Ash content was calculated as 12.4% using a Neycraft Vulcan 3-550 burnout furnace at 550 °C, consistent with literature values for dried *Avicennia marina* leaves^34,35^. This ash value aligns well with previously reported values of 7.17–15.3% for dried *Avicennia marina* leaves across various geographical locations^34^.

#### Characterization of CuO NPs

X-ray Diffraction was conducted for crystalline phase identification and structural analysis of CuO NPs both in their pristine state and following platinum drug loading using a Bruker D8 Discover Diffractometer. This analysis provided insights into the crystallographic properties and phase purity of the materials before and after drug loading. Fourier Transform Infrared (FTIR) Spectroscopy was performed to determine the chemical functional groups on the unloaded CuO NPs and the two examined platinum-loaded formulations using a Nicolet 380 FT-IR Spectrometer operating within the spectral range of 500–4000 cm⁻¹. FTIR spectroscopy served as a preliminary verification method for successful drug incorporation by enabling comparative analysis of characteristic absorption bands between the loaded and unloaded samples. Zeta Potential measurements were performed to determine the surface charge of the CuO NPs by means of dynamic light scattering using a Zetasizer Nano ZS90 instrument (Malvern Instruments, UK) maintained at 25 °C. Analysis was performed on samples prepared as 1:10 (v/v) dilutions in distilled water (pH ≈ 7.4), yielding a measured zeta potential value of − 13.9 mV, indicating a slightly negative surface charge. Following drug loading, the zeta potential values were measured as − 11.3 mV (cisplatin@green-CuO) and − 12.1 mV (oxaliplatin@green-CuO). The slight decrease in negative surface charge relative to bare CuO NPs (− 13.9 mV) is consistent with partial occupation of anionic surface oxygen sites by Pt(II) complexes, while the values remain sufficient for moderate colloidal stability in aqueous suspension^36,37^. Dynamic light scattering (DLS) analysis revealed a hydrodynamic diameter of 161 nm with polydispersity index (PDI) of 0.28 for the bare CuO NPs, suggesting a moderately narrow size distribution consistent with reasonable colloidal stability in aqueous suspension.^38^ Following drug loading, the hydrodynamic diameter increased to 254 nm (PDI = 0.32) for cisplatin@green-CuO and 267 nm (PDI = 0.35) for oxaliplatin@green-CuO. The textural properties of CuO NPs were evaluated before and after loading with Pt(II) drugs by measuring nitrogen adsorption-desorption isotherms at 77 K with a Micromeritics ASAP 2020. Samples were vacuum degassed at 40 °C for 6 h. The Brunauer-Emmett-Teller (BET) and the Barrett-Joyner-Halenda (BJH) approaches were then used to determine specific surface areas and pore size distributions.

#### Determination of loading capacity and entrapment efficiency

A quantified amount of cisplatin (12 mg) and oxaliplatin (35 mg), determined by their solubility profiles, were each dissolved in 5 mL of distilled water^39,40^. CuO NPs were then added to these solutions at a mass ratio of 2:1 (nanoparticle to drug). The mixtures were continuously stirred and left to incubate at ambient temperature for 48 h to facilitate drug adsorption onto the nanoparticles. Following the incubation period, the samples underwent centrifugation at 4000 rpm for 30 min to separate the nanoparticles from the solution. Carefully collected supernatants were subjected to quantitative analysis using a Cary 3500 UV-Visible spectrophotometer (model G9864A, Mulgrave VIC 3170, Australia). Absorbance readings were obtained within the 200–230 nm wavelength range to determine drug concentrations. Quantification was performed against five-point calibration curves specific to each drug. Entrapment efficiency (EE%) and the loading capacity were calculated using the following equations (Eq. 1 and Eq. 2):

1 $$\:Entrapment\:Efficiency\:\left(EE\%\right)=\:\frac{Amount\:of\:Substance\:Entrapped}{Total\:Substance\:Amount}\:\times\:\:100$$

2 $$\:Loading\:Capacity=\:\frac{\left(Initial\:drug\:concentration\:-\:Final\:drug\:concentration\right)\times\:\:Volume\:of\:solution\:}{mass\:of\:the\:adsorbent}\:\:$$

## Results and discussion

In this work, CuO NPs were produced by a green approach using *Avicennia marina* (mangrove) leaf extract and fully characterized, while their drug-loading performance was examined. Notably, all analyses pertaining to the green-derived CuO NPs, including their structural and surface characteristics before and after loading with cisplatin and oxaliplatin, were conducted. The investigation centers on the practical advantages and physicochemical features afforded by phytogenic CuO NPs, with particular attention to their mesoporous architecture and crystalline integrity as revealed by XRD, SEM, BET, FTIR, and UV–Vis characterization. The loading behavior of cisplatin and oxaliplatin is systematically examined, emphasizing the role of the unique organic capping environment imparted by the plant extract in modulating drug-nanoparticle interactions. The release kinetic profiles of both drugs were also investigated.

### Crystallinity

**Fig. 2:**
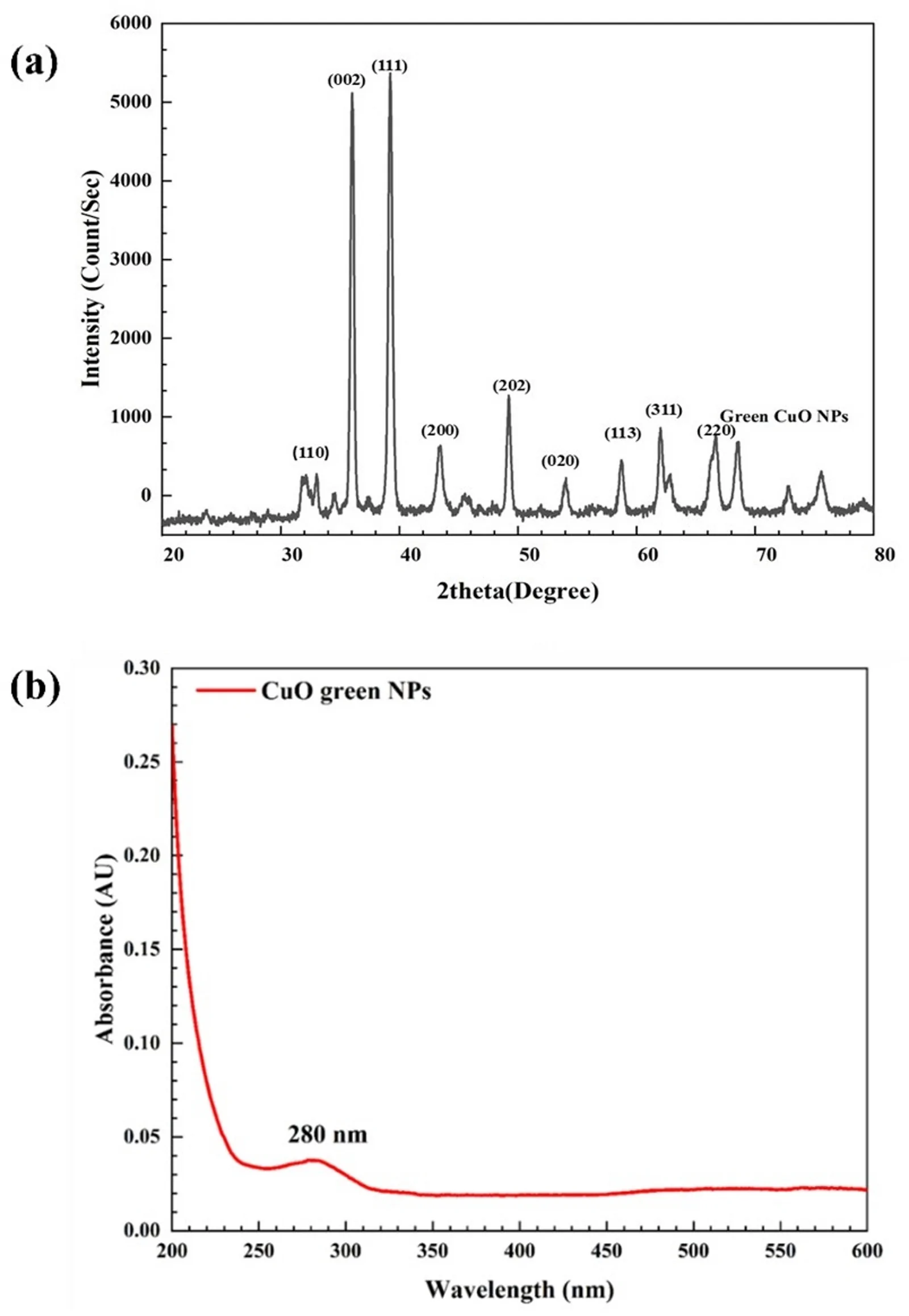
(**a**) XRD pattern, and (**b**) UV-Vis spectrum of green CuO NPs.

The X-ray diffraction (XRD) pattern of green-synthesized CuO NPs exhibits prominent and well-defined peaks at 2θ values of 36°, 39°, 43.8°, 49.2°, 50.9°, 54°, 58.7°, 62°, 66.6°, 68.5°, 72.8°, and 75.5° (shown in Fig. 2a). These diffraction peaks correspond to the characteristic lattice planes of monoclinic CuO, in agreement with JCPDS Card No. 48-1548. The sharpness and intensity of the peaks indicate a high degree of crystallinity. No additional insignificant peaks or impurities are observed, confirming the purity of the synthesized CuO nanoparticles. The major peaks, particularly those at 36° and 39°, can be indexed to the (002) and (111) planes, respectively. The other reflections correspond to crystal planes such as (200), (202), (020), (211), and (–202), which are typical for monoclinic CuO and are in excellent agreement with the literature.^37,41^ Minor other peaks could be assigned to trace Cu₂O (JCPDS 05-0667), a known minor phase in plant-mediated CuO synthesis arising from incomplete oxidation. The relative intensity of these peaks is very minor, indicating that the synthesized material is predominantly phase-pure monoclinic CuO.^42^ Beyond crystallite size, the interplanar spacings were determined to further verify the crystalline phase. Using Bragg’s law, the experimental d-spacings of the three principal reflections were calculated as 0.249 nm for the (002/(111)) reflection at 2θ = 36.02°, 0.229 nm for the (111) reflection at 2θ = 39.23°, and 0.185 nm for the (202) reflection at 2θ = 49.20°. These values are in close agreement with the standard d-spacings of monoclinic CuO (JCPDS Card No. 48-1548). The close correspondence between the experimental and standard lattice parameters further validates the successful formation of phase-pure crystalline CuO nanoparticles.^43,44^ In the drug-loaded composites, the absence of additional diffraction peaks suggests that cisplatin and oxaliplatin are highly dispersed or amorphous on the CuO surface rather than forming separate crystalline phases, consistent with the surface-adsorption mechanism supported by FTIR and BET analysis.

### Optical properties

UV-Vis spectroscopic analysis was employed to investigate the optical properties of the green-synthesized CuO NPs. The UV-Vis absorption spectrum of the CuO NPs, shown in Fig. 2b, exhibits a characteristic absorption peak at 280 nm. This absorption peak in the 280 nm range is consistent with previously reported values for biogenically synthesized copper oxide nanoparticles and confirms the successful formation of nanoscale CuO particles^45,46.^

### Surface morphology and elemental analysis

**Fig. 3:**
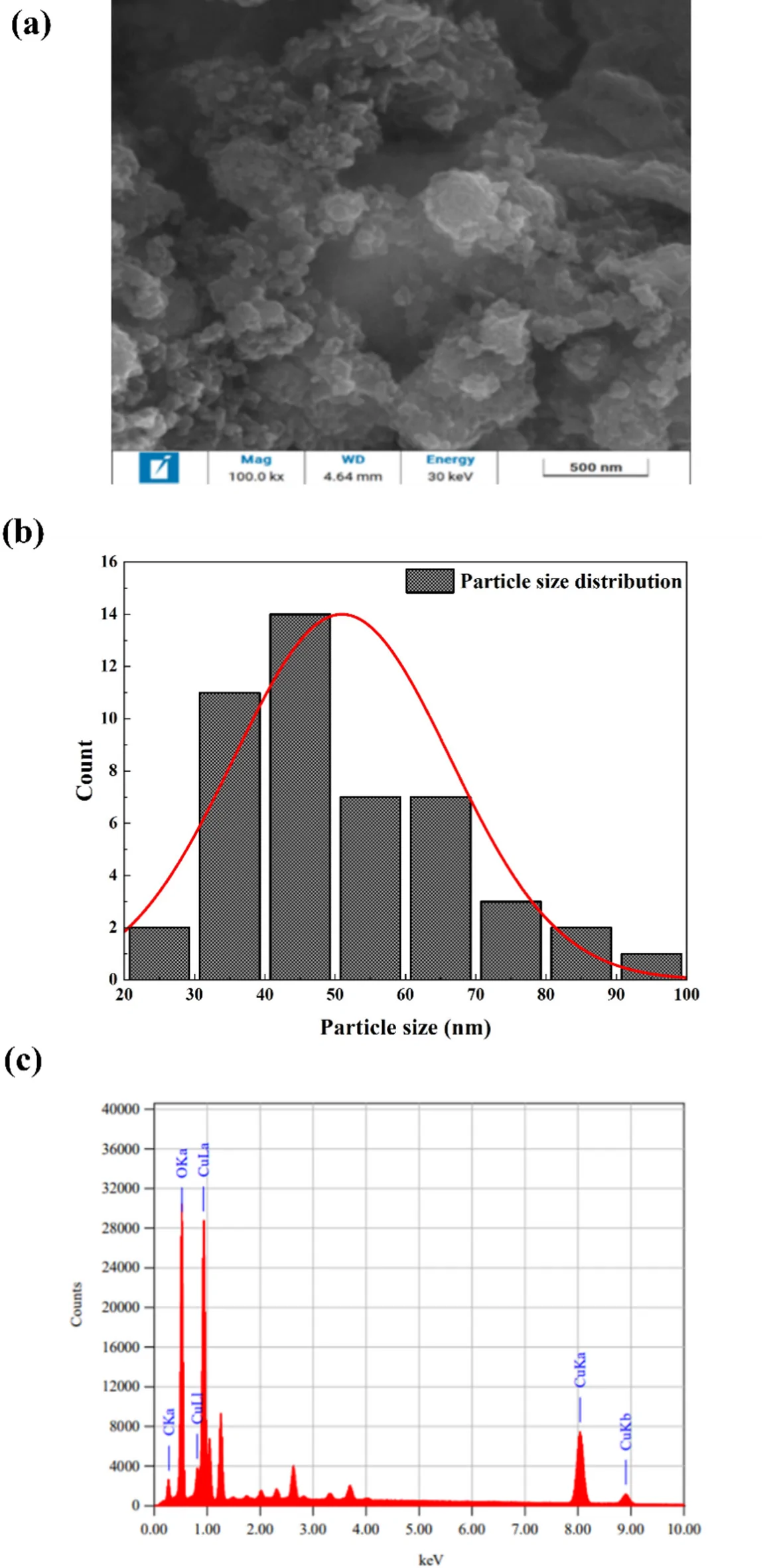
(**a**) SEM image, (**b**) Particle size distribution, and (**c**) EDX analysis of the green CuO NPs.

Scanning Electron Microscopy (SEM) was used to assess the shape and size distribution of green-synthesized CuO NPs. The SEM image shows that the particles have a quasi-spherical shape with some agglomerations. Individual nanoparticle size estimation from the SEM image (shown in Fig. 3a) indicates particles ranging from 35 to 87 nm (Fig. 3b), which is characteristic of plant-mediated green synthesis. Surface-capping by phytochemicals from *Avicennia marina* extract facilitates particle stabilization but also contributes to non-uniform growth and secondary clustering.^47,48^ After loading, the cisplatin@green-CuO and oxlaiplatn@green-CuO SEM micrographs (shown in Figure S1 and S2) confirm retention of quasi-spherical morphology and do not show significant particle agglomeration or morphological change after loading, supporting the structural integrity required for biomedical use.

Energy Dispersive X-ray (EDX) spectroscopy was employed as a complementary analytical technique to confirm the elemental composition and purity of the green-synthesized CuO nanoparticles. The EDX spectrum (Fig. 3c) displays characteristic peaks corresponding to copper (Cu) and oxygen (O) elements, which are the primary constituents of the synthesized nanoparticles. Quantitative analysis of the EDX data revealed the atomic percentages of the major elements present in the samples. The EDX spectrum revealed Cu at 41.86 atomic %, O at the expected level for CuO stoichiometry, and C at 11.09 atomic % from phytochemical capping agents of the *Avicennia marina* leaf extract. The characteristic Cu K-shell (8.0 keV) and O K-shell (0.5 keV) peaks confirmed successful CuO formation without extraneous metallic impurities. The Cu composition is consistent with literature values for green-synthesized CuO nanoparticles, which typically range from 27.62% to 64.19% depending on synthesis conditions.^49–51^ To provide direct elemental evidence for the successful loading of the platinum therapeutics onto the carrier, EDX analysis was additionally performed on the drug-loaded nanocomposites. The EDX spectra of the cisplatin@green-CuO and oxaliplatin@green-CuO samples (Figure S3 and S4) display distinct platinum signals alongside the chlorine signal arising from the cisplatin ligands. Quantitative analysis confirmed platinum incorporation, with Pt detected at 12.42 atomic % for cisplatin@green-CuO and 6.90 atomic % for oxaliplatin@green-CuO in the two loaded formulations, respectively. These Pt-specific peaks are absent in the spectrum of the bare green-CuO NPs, confirming that the platinum drugs were effectively immobilised on the nanoparticle surface and providing complementary elemental evidence to the FTIR coordination data. EDX elemental data are reported as atom% to avoid mass-based overestimation arising from platinum’s relatively high atomic mass; the Pt atom% values of 12.42% (cisplatin@green-CuO) and 6.90% (oxaliplatin@green-CuO) are consistent with the loading capacities determined by UV-Vis spectrophotometry.

### Spectral analysis and nanoparticle-drug interactions

**Fig. 4:**
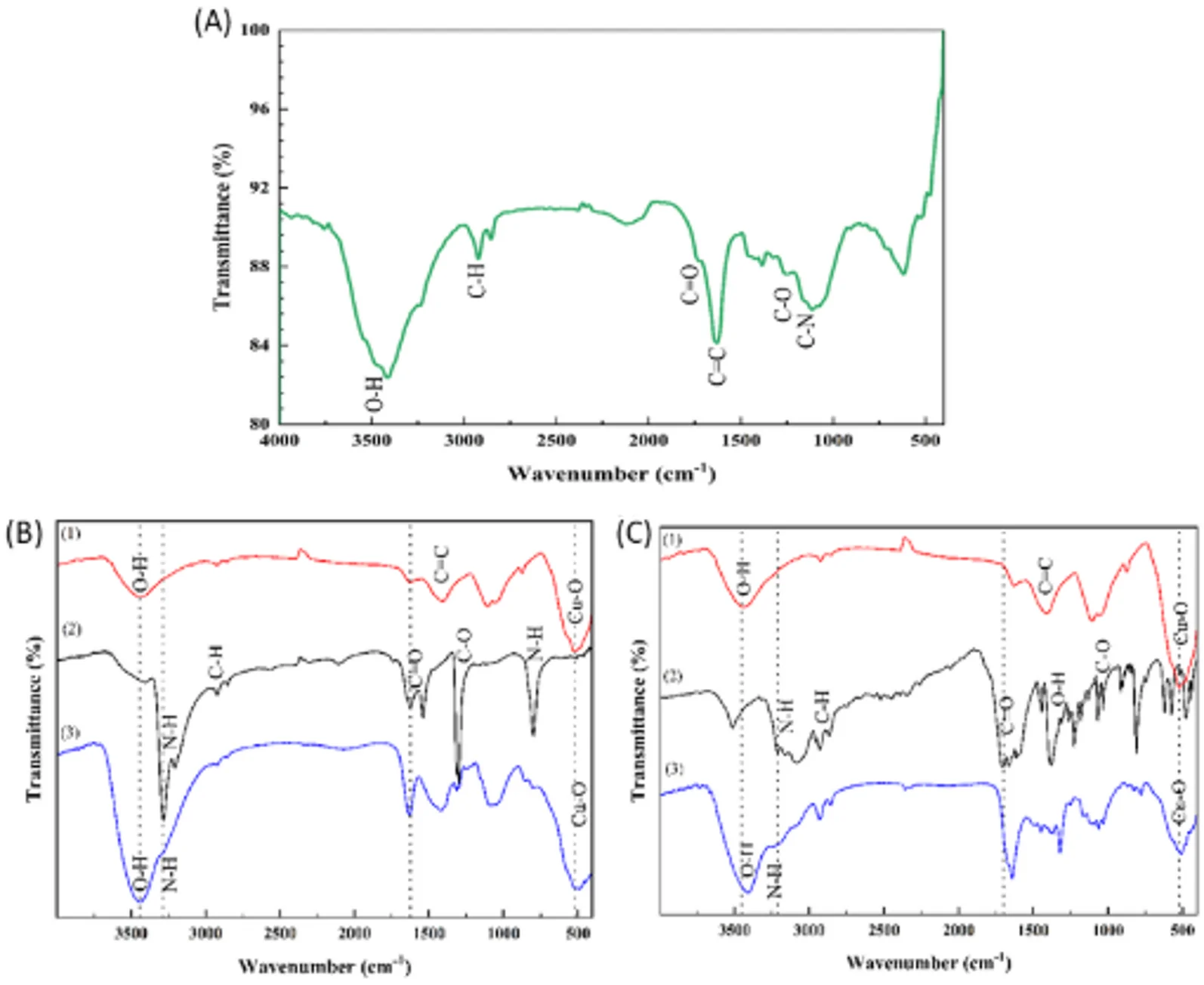
(**A**) FTIR spectrum of Mangrove Leaves: (**B**) FTIR spectra of the free CuO NPs (red line1), free cisplatin (black line 2) and the cisplatin@green-CuO complexes (blue line 3); (**C**) FTIR spectra of the free CuO NPs (red line 1), the free oxaliplatin (black line 2) and the oxaliplatin@green-CuO complexes (blue line 3).

To gain more insights into the interactions taking place between the CuO NPs and the tested drugs, the main functional groups on both the nanoparticle and the drug are determined using FTIR analysis. In addition, the main functional groups of the mangrove leaf extract are also analyzed since these groups cap the formed green CuO NPs. The FTIR spectrum of the mangrove leaf extract exhibits distinct absorption bands ( Fig. 4A), indicating the presence of several bioactive chemicals responsible for CuO nanoparticle reduction and stability.^19^ The large absorption peak at 3400–3500 cm⁻¹ is due to O-H stretching vibrations of hydroxyl groups in phenolic compounds and alcohols. The peaks at 2800–3000 cm⁻¹ correspond to C-H stretching vibrations of aliphatic molecules. The absorption band at 1600–1650 cm⁻¹ represents C = C stretching vibrations in aromatic compounds and C = O stretching in carbonyl groups. Peaks in the fingerprint area (1000–1500 cm⁻¹) show C-O stretching vibrations in alcohols and phenols.^9,52,53^ These features confirm the presence of polyphenols, flavonoids, and other oxygenated metabolites, which act as reducing and capping agents in the green synthesis process. ^54–57^

The FTIR spectrum of the green-synthesized CuO NPs (Fig. 4B, line1; Fig. 4C, line1) preserves a broad O–H band in the 3400–3500 cm⁻¹ region and C–O/C–O–C bands in the fingerprint region, indicating that phytochemicals from the mangrove extract remain adsorbed on the nanoparticle surface and constitute an organic capping layer. In addition, new bands in the low-wavenumber region around 500–600 cm⁻¹ are assigned to Cu–O lattice vibrations, confirming the formation of crystalline CuO. Compared to the pure extract, the relative attenuation and slight shifts of some C = O/C–O bands upon nanoparticle formation suggest that these functional groups participate in coordination to Cu²⁺ and stabilization of the CuO surface.^3,8,58,59^.

The FTIR spectra of the free drugs (Figs. 4B, line 2 and Fig. 4C, line 2) exhibit characteristic bands that serve as markers for cisplatin and oxaliplatin. For cisplatin, N–H stretching vibrations of the coordinated amine ligands appear in the 3200–3400 cm⁻¹ region, accompanied by N–H bending and Pt–N/Pt–Cl related bands at lower wavenumbers. Oxaliplatin shows N–H stretching from the diaminocyclohexane ligand together with C = O and C–O stretching bands associated with the oxalate moiety in the 1600–1750 cm⁻¹ and 1000–1300 cm⁻¹.^60–63^

Upon loading cisplatin and oxaliplatin onto the green CuO NPs, the FTIR spectra of the resulting nanocomposites (Figs. 4B, line 2 and Fig. 4C, line 2) display a superposition of features from both the CuO core and the drug molecules. The O–H and N–H stretching bands become broader and shift slightly relative to the free components, indicating the establishment of hydrogen bonding or coordination interactions between the drug ligands, the phytochemical capping layer, and surface Cu/O sites. The Cu–O stretching bands in the 500–600 cm⁻¹ region remain clearly visible with only minor changes in position or intensity, demonstrating that the CuO lattice retains its structural integrity after drug loading. Importantly, drug-specific bands (amine N–H and carbonyl/oxalate vibrational modes) are still present in the loaded systems with reduced intensity and partial shifts, consistent with successful immobilization of cisplatin and oxaliplatin on the nanoparticle surface.^60,64–66^ The FTIR spectra of both drug-loaded composites confirmed the presence of all three components. The Cu–O stretching vibration (~ 530 cm⁻¹) was retained in both samples, confirming preservation of the CuO structure after loading. Bands at 1000–1300 cm⁻¹ and the broad O–H stretch at ~ 3400 cm⁻¹ are attributed to the phytochemical capping layer. Drug incorporation was confirmed by N–H stretching bands at 3200–3400 cm⁻¹ from the amine ligands of both drugs, and by a C = O stretching band at 1680–1720 cm⁻¹ in the oxaliplatin@green-CuO spectrum assigned to the oxalate ligand. Shifts in the Cu–O band position relative to bare CuO NPs are consistent with Pt–Cu surface coordination. ^60^

These FTIR data support the significance of mangrove leaf extract in nanoparticle formation and stability. The existence of both drug and nanoparticle characteristic bands, as well as changes suggesting significant interactions, confirms that cisplatin and oxaliplatin were successfully loaded onto green-synthesized CuO NPs. After drug loading, the CuO structure maintains its integrity, indicating that these nanocomposites have the potential for drug delivery applications.

### Surface characteristics and nanoparticle-drug interactions

**Fig. 5:**
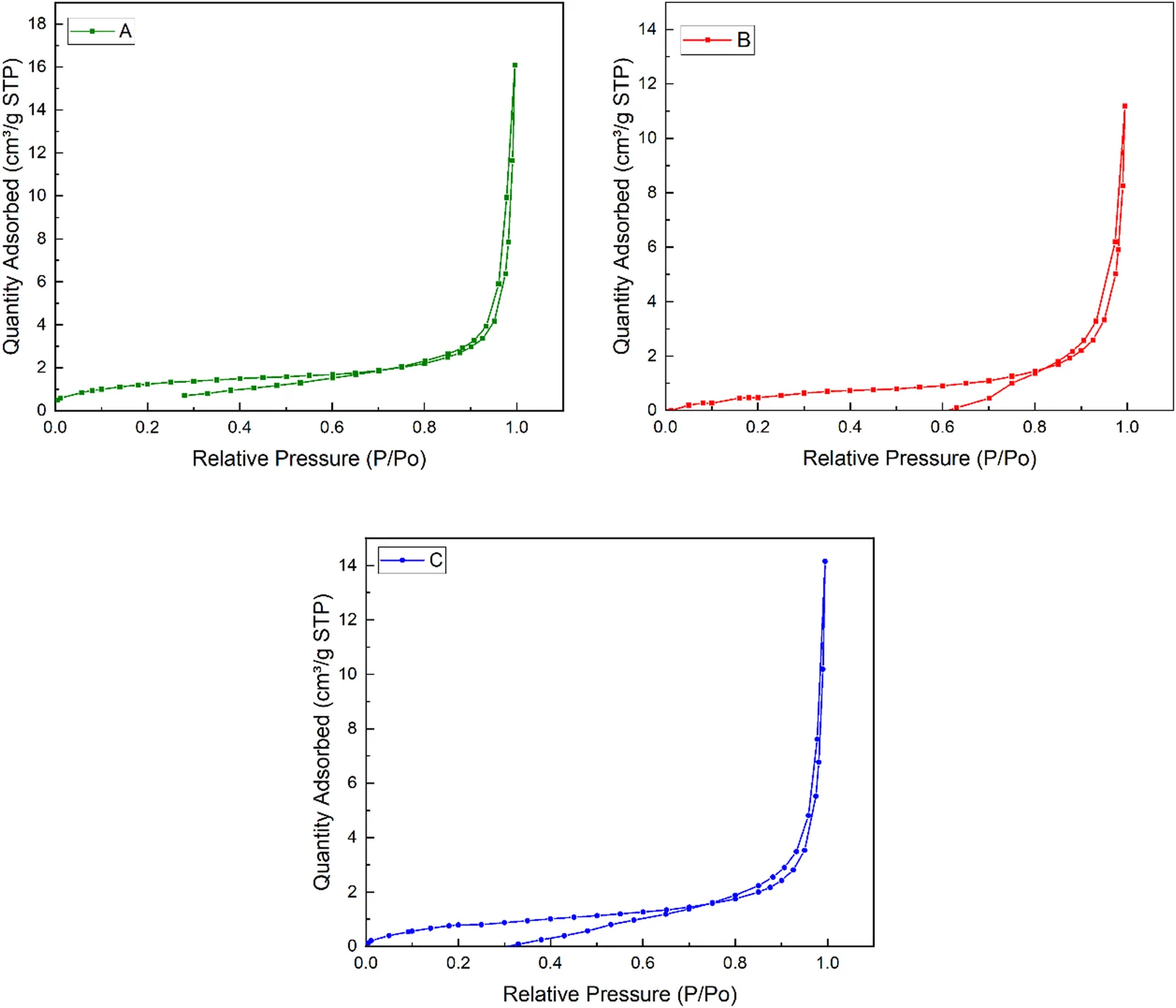
Nitrogen adsorption-desorption isotherms for (**A**) free green CuO NPs, (**B**), cis@green-CuO, and (**C**) oxa@green-CuO.

The porosity and surface properties of the green-synthesised CuO NPs are investigated by studying their nitrogen adsorption–desorption isotherms (Fig. 5, panel A), as well as their isotherms after loading cisplatin, cis@green-CuO (panel B), and oxaliplatin, oxa@green-CuO (panel C). All isotherms exhibit type IV, which are characteristic of mesoporous materials, with a hysteresis loop caused by capillary condensation within the pores. ^36^ Mesoporosity is generally advantageous for drug loading, as it provides more accessible sites for drug molecules to adsorb onto the surface of the CuO NPs via multilayer pore filling mechanisms, and thus can consequently improve the drug loading efficiency.

**Fig. 6:**
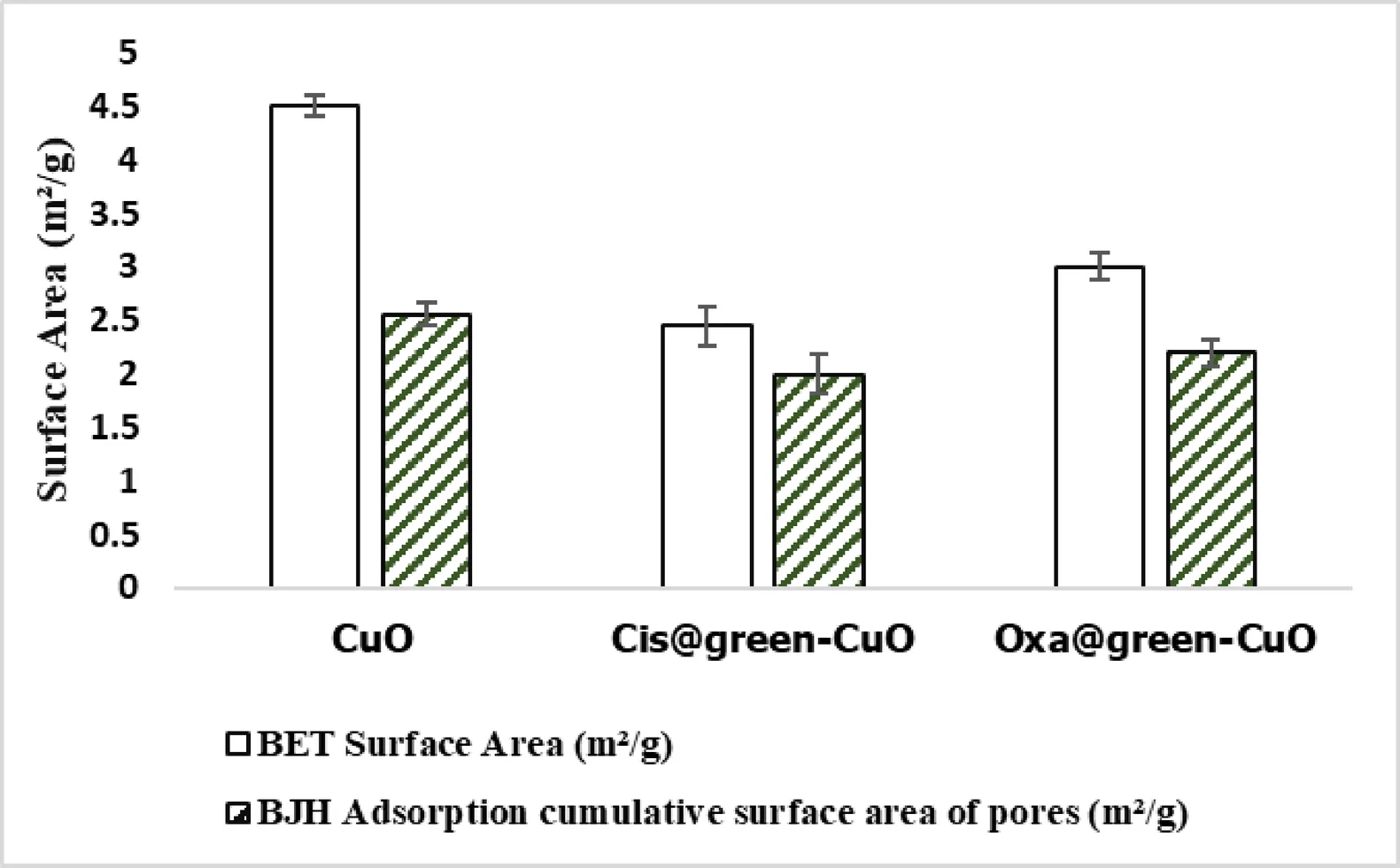
The BET surface area of the green CuO NPs before and after loading the Pt-drugs.

The BET surface area and the BJH adsorption cumulative surface area of the pores of CuO NPs before and after loading the cisplatin and the oxaliplatin drugs are shown in Fig. 6. The surface area of pores decreased when the cisplatin and the oxaliplatin were loaded on the surface of the copper oxide nanoparticles.

The BET surface area analysis reveals that the green-synthesized CuO NPs exhibit an initial surface area of 4.5 m²/g, lower than the 15 m²/g reported for chemically synthesized CuO nanoparticles using hydrothermal methods^26^, this can be attributed to the organic capping agents from the plant extract that may partially block surface sites. Upon drug loading, both cisplatin and oxaliplatin cause significant reductions in surface area to 2.45 m²/g and 3.0 m²/g, respectively, indicating successful drug adsorption onto the nanoparticle surface. The difference between BET total surface area and BJH mesopore surface area is expected as the BET quantifies the total accessible surface (external and all pore surfaces), while BJH primarily measures the mesopore surface area from the Kelvin-equation, while ignoring the micropore contribution.^36^ For materials with bimodal (micro and meso) porosity, as seen here, the BJH value is necessarily lower than BET.^36^ All N₂ adsorption–desorption measurements were performed in duplicate; reported values represent the mean of two independent measurements.

**Fig. 7:**
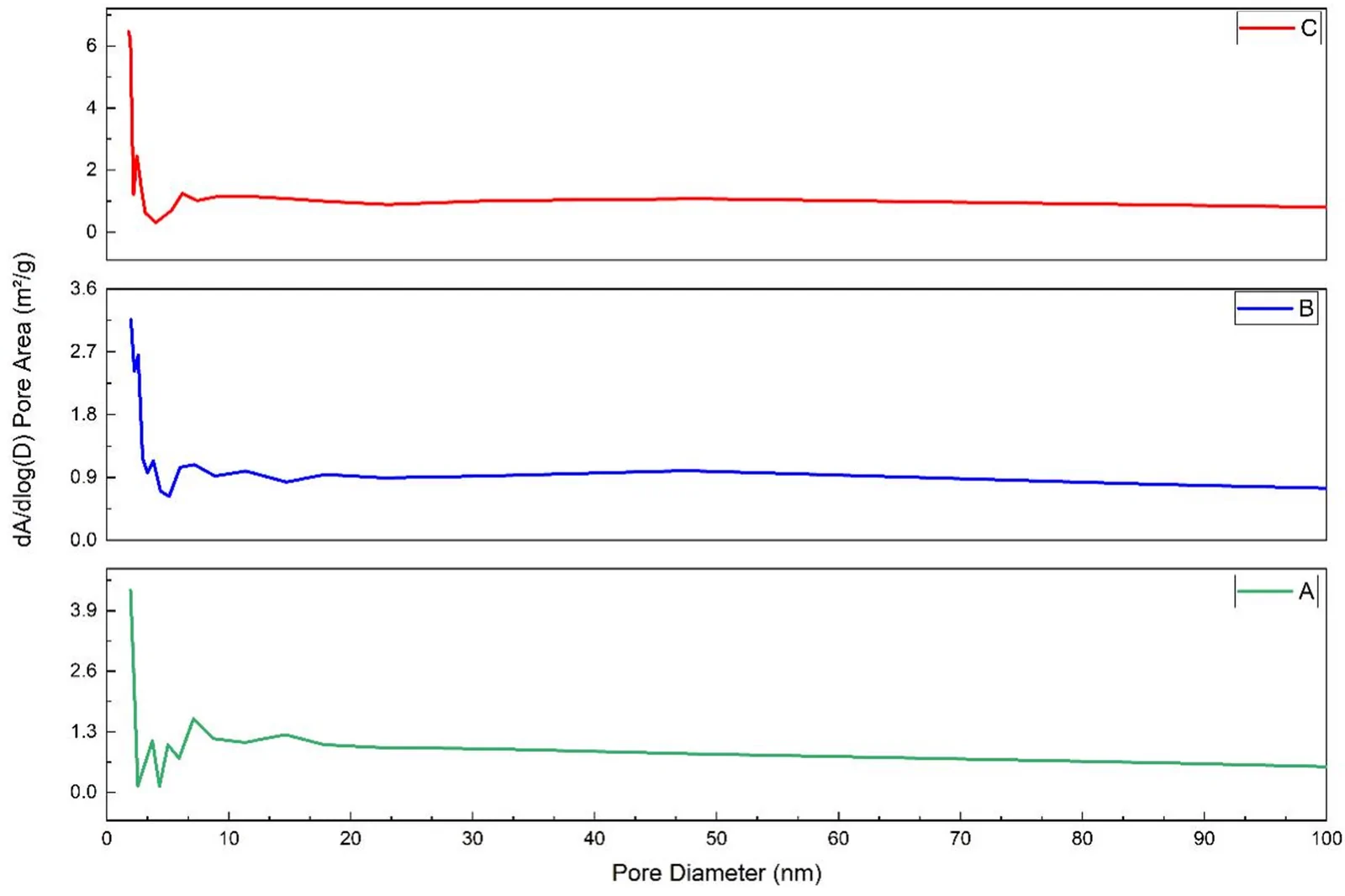
BJH pore size distribution expressed as adsorption dA/dlog (**D**) Pore Area, for the free green-CuO NPs (**A**), cis@green-CuO (**B**), and oxa@green-CuO (**C**).

**Fig. 8:**
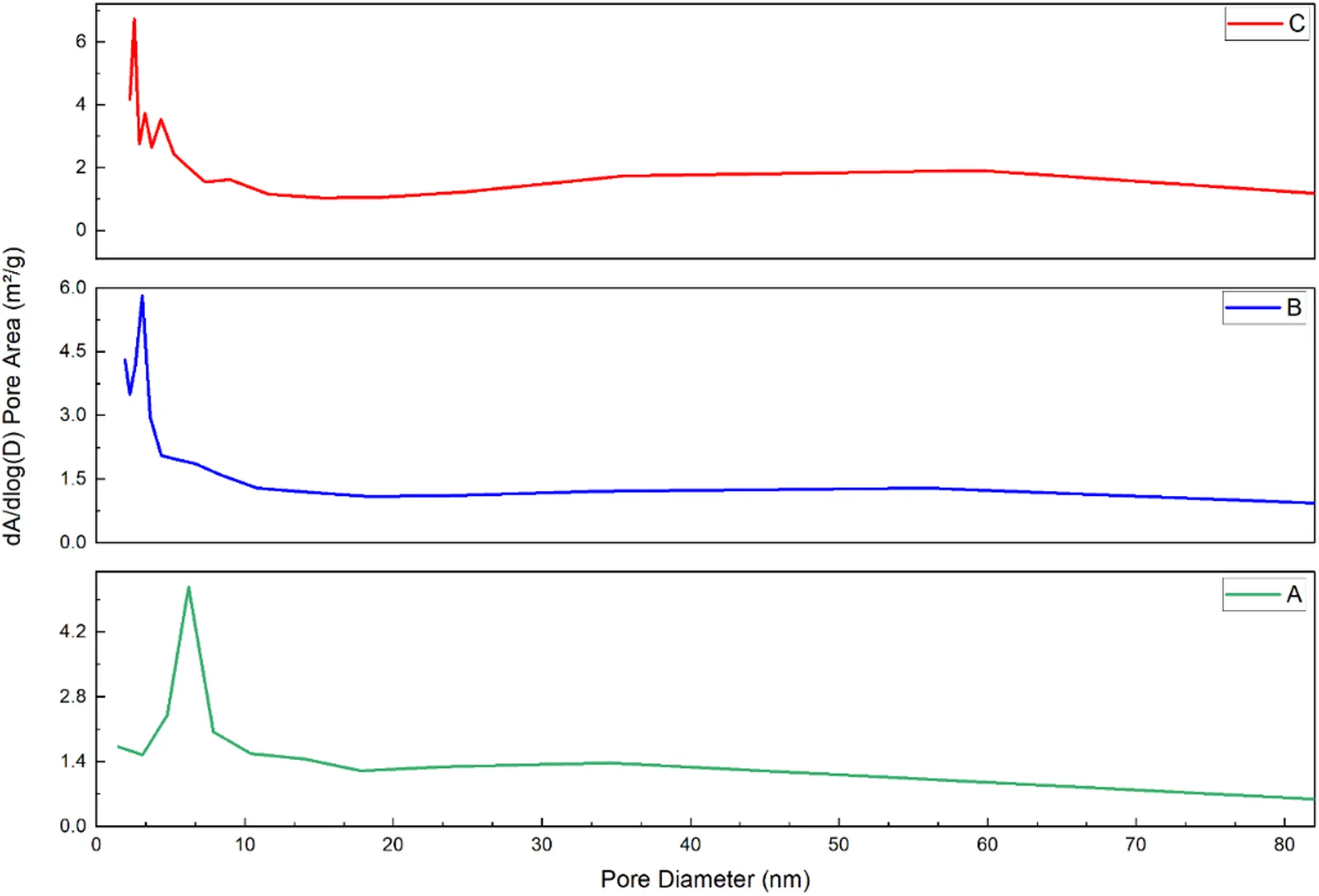
BJH Desorption dA/dlog (**D**) Pore Area for the free green-CuO NPs (**A**), cis@green-CuO (**B**), and oxa@green-CuO (**C**).

The BJH pore size distribution profiles of CuO NPs, cis@green-CuO, and oxa@green-CuO as estimated from the adsorption (Fig. 7) and desorption (Fig. 8) reveal predominantly mesoporous characteristics with pore diameters spanning 2–50 nm, consistent with the type IV isotherms and advantageous for drug molecule adsorption. Notably, both figures show a small population of micropores (< 2 nm) alongside the dominant mesopore fraction, evidenced by the low-diameter tail in the dA/dlog(D) pore area distributions; this bimodal porosity arises from phytochemical templating during green synthesis and enhances surface accessibility. Upon cisplatin and oxaliplatin loading, the profiles exhibit shifts toward narrower mesopore distributions with reduced micropore contributions, indicating partial occupancy of smaller pores and external surface sites by the drugs without collapsing the mesoporous architecture.^67,68^.

**Fig. 9:**
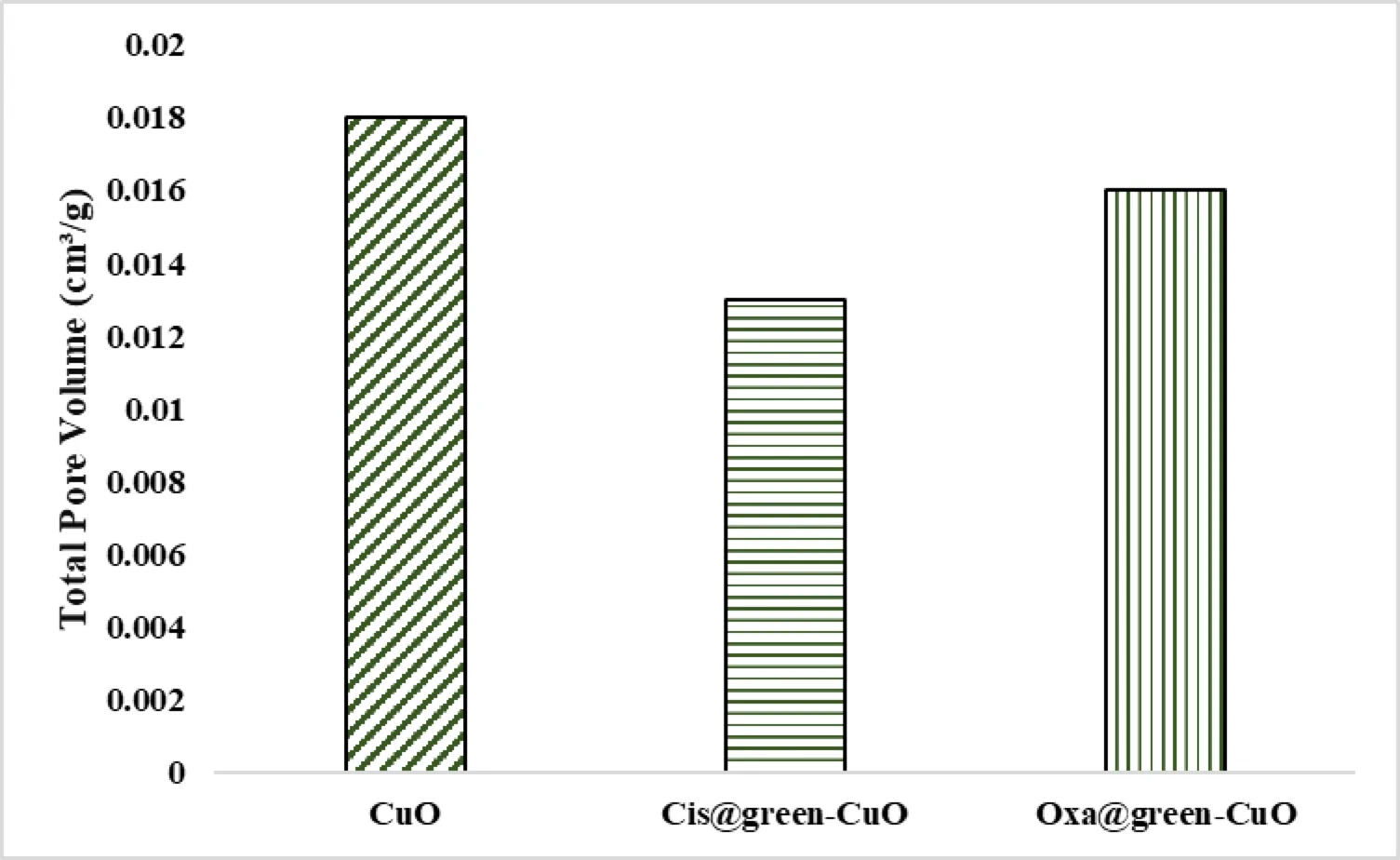
Total pore volume of the green CuO NPs before and after loading of Pt-drugs.

The total pore volume was also analysed for the free CuO NPs before and after loading with the Pt(II)-drugs as shown in Fig. 9.

The total pore volume of green-synthesized CuO nanoparticles is 0.018 cm³/g substantially lower than the 0.06 cm³/g reported for chemically synthesized nanoparticles^26^, reflecting partial pore occlusion by the phytochemical capping layer from *Avicennia marina* extract, and drug loading results in minimal changes in pore volume, indicating that drug molecules preferentially adsorb on external surfaces rather than penetrating deeply into the pore structure of the green-synthesized nanoparticles. The pore volume of the nanoparticles was reduced to 0.013 cm³/g after loading with cisplatin and to 0.016 cm³/g after loading with oxaliplatin. Cisplatin causes a greater pore volume reduction due to its stronger adsorption affinity for CuO surfaces.^26^.

### Thermal stability of the loaded nanoparticles

Thermogravimetric analysis (TGA) was performed to evaluate the thermal stability and degradation profiles of the drug-loaded CuO NPs. The TGA curves (Figure S5) show the weight loss of cisplatin@green CuO NPs and oxaliplatin@green CuO NPs as a function of temperature from ambient to 800 °C. Cisplatin@green CuO NPs exhibited superior thermal stability with approximately 17% weight loss up to 800 °C, while oxaliplatin@green CuO NPs showed approximately 22% weight loss over the same temperature range. The initial gradual weight loss in both samples (ambient to ~ 200 °C) is attributed to removal of moisture and residual water from the nanoparticle surface. Subsequent weight loss occurring between 200 and 400 °C corresponds to the thermal decomposition and desorption of phytochemical capping agents and weakly adsorbed drug molecules from the nanoparticle surface.^69^ The differential thermal profiles reflect the different binding affinities and drug-nanoparticle interactions of cisplatin and oxaliplatin.

### Drug loading efficiency of the nanoparticles

UV-Vis spectroscopic analysis was conducted to quantify the adsorption of Pt (II)-based therapeutic agents onto CuO nanoparticle surfaces. The characteristic absorption spectra of cisplatin and oxaliplatin were monitored within the wavelength range of 200–240 nm, consistent with previously reported literature.^70–72^ The UV–Vis absorption spectra of the free drugs and their CuO-loaded complexes are compared in Figure S6. Free oxaliplatin and free cisplatin each display their characteristic absorption in the 200–260 nm region. Upon loading onto the green-CuO NPs, both oxaliplatin@green-CuO and cisplatin@green-CuO show a marked attenuation of absorbance relative to the corresponding free drug at equal nominal concentration, consistent with depletion of free drug from solution through adsorption onto the nanoparticle surface. This decrease in the supernatant absorbance is the basis for the quantitative determination of entrapment efficiency and loading capacity reported below. Standard calibration curves were established for each platinum compound, enabling the determination of entrapment efficiency (EE%) and drug loading capacity. Entrapment efficiency represents the fraction of drug successfully incorporated within the nanocarrier matrix relative to the initial drug concentration employed during synthesis. This parameter serves as a critical quality indicator for drug delivery systems, as it directly influences the therapeutic efficacy and targeted delivery performance of the nanocarrier.^73^ The loading capacity quantifies the maximum drug payload achievable per unit mass of nanoparticles, providing essential information for dosage optimization. Both EE% and drug loading capacity were calculated using established mathematical relationships given in the experimental section. Cisplatin exhibited an EE% of 28.3% and a drug loading capacity of 56 mg/g, while oxaliplatin showed an EE% of 17% and a drug loading capacity of 34 mg/g. These values are lower than those obtained with commercial CuO nanoparticles (15 m²/g surface area) reported in our previous study (EE% cisplatin 52%, and oxaliplatin 38%), which is understandable given the green NPs’ lower surface area (4.5 m²/g).

The observation that cisplatin exhibits higher loading efficiency compared to oxaliplatin is consistent with theoretical predictions from density functional theory (DFT) calculations reported for CuO nanoparticles synthesized via chemical methods.^26^ Using DFT simulations under periodic boundary conditions, it was demonstrated that cisplatin exhibits the strongest interaction with the CuO (111) surface, with an adsorption energy (Eads) of − 3.0 eV, compared to oxaliplatin and nedaplatin.^26^ For green CuO NPs, FTIR evidence of residual –OH from mangrove polyphenols and –NH₂/–OH drug ligands supports additional H-bonding interactions with the hydroxylated surface (as modeled for OH-terminated CuO in our prior work), complementing direct Pt–Cu binding and explaining efficient loading despite partial site passivation. ^26^The computational study revealed that all cisplatin and oxaliplatin drugs preferentially coordinate with tri-coordinated surface copper atoms, but cisplatin’s molecular structure, featuring two chloride and two ammonia ligands, facilitates more intense contact with the oxide surface. This enhanced binding affinity, which translates experimentally to strong drug loading, is attributed to the favorable geometry and electronic configuration enabling stronger Pt–Cu coordination. The present experimental findings with green-synthesized CuO NPs corroborate these theoretical predictions, demonstrating that the preferential binding of cisplatin over oxaliplatin persists regardless of the synthesis method of the NPs.

### Drug release kinetic modeling

**Fig. 10:**
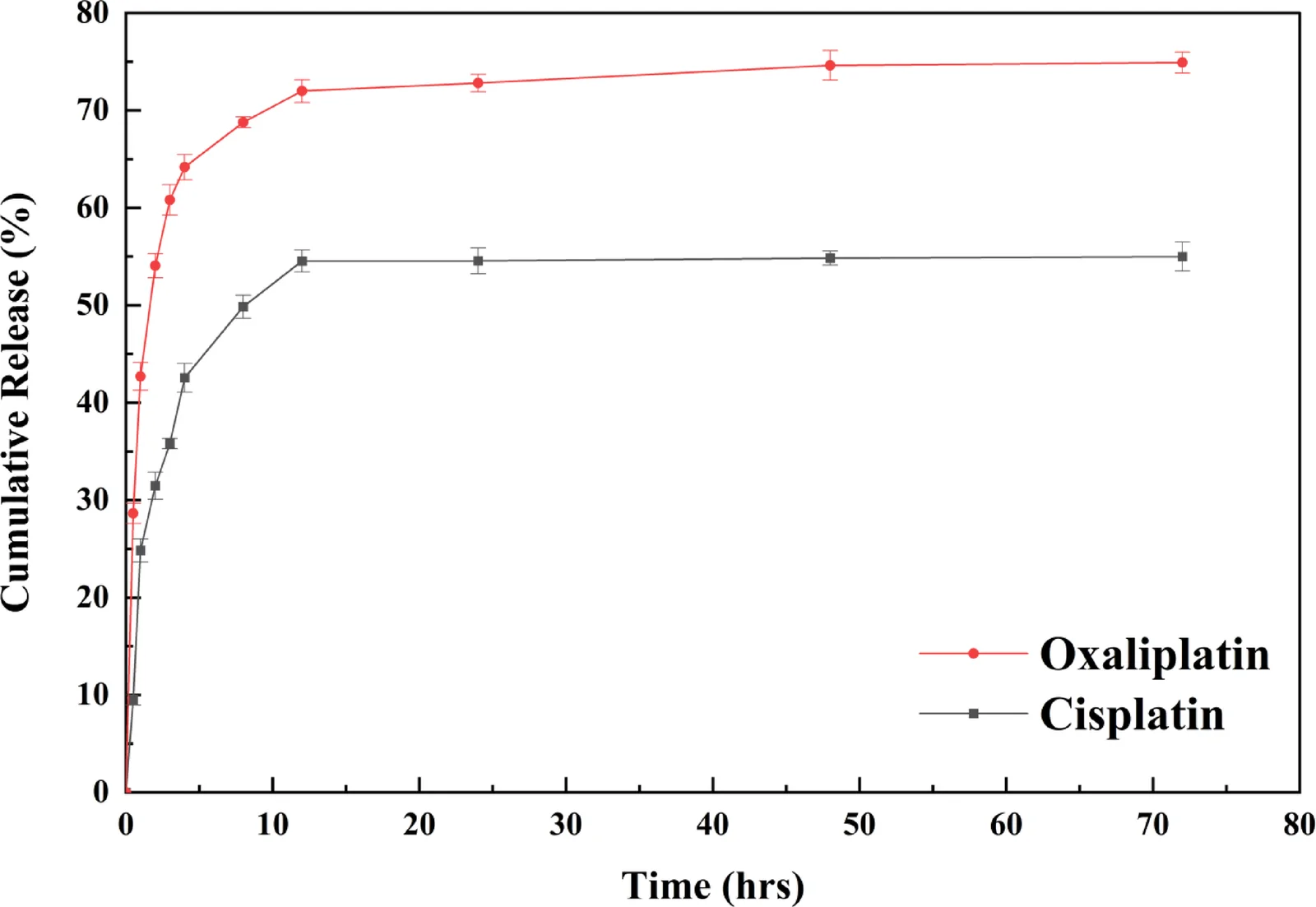
Drug release profiles of cisplatin and oxaliplatin, Data are presented as mean ± SD (*n* = 3).

The in vitro drug release profiles of cisplatin and oxaliplatin from mangrove-derived green CuO nanoparticles (Fig. 10), conducted under simulated physiological conditions (pH 7.4 phosphate buffer saline, 37 °C), demonstrate sustained and controlled release over 80 h, characteristic of surface-adsorption-based nanocarriers. Cisplatin exhibits a biphasic pattern with an initial burst release (20% in the first 10 h) due to weakly bound surface molecules, followed by slower sustained release reaching 70% cumulative release by 20 h; oxaliplatin shows a similar but slightly faster profile, achieving about 80% release over the same period, likely reflecting its weaker adsorption energy compared to cisplatin. In comparison, commercial CuO nanoparticles (chemically synthesized) loaded with the same drugs displayed more pronounced initial bursts and 32% cisplatin release after 72 h, attributed to stronger Pt–Cu coordination (E_ads = − 3.0 eV) and less organic capping that retards diffusion. ^26^Overall, both green and commercial CuO systems exhibit sustained release profiles originating mainly from surface adsorption, yet the green CuO NPs achieve a substantially more rapid cumulative release, reaching 70% cisplatin within 20 h versus only 32% for commercial CuO NPs after 72 h. To further elucidate the release mechanism, the experimental data were fitted to zero-order, first-order, Higuchi, and Korsmeyer–Peppas kinetic models, with the corresponding linear plots presented in the Supplementary Information (Figure S7). The zero-order model showed poor correlation for both drugs, indicating that release is not constant over time. For cisplatin, the first-order model provided the best description of the release data (R² = 0.91), consistent with a concentration-dependent release process. For oxaliplatin, the Higuchi model gave the closest fit (R² = 0.81), exceeding the first-order fit and indicating that release is predominantly governed by diffusion from the mesoporous CuO matrix. The comparatively moderate correlation coefficients reflect the complex, multi-phasic release behavior typical of surface-adsorption-based nanocarriers, for which simple single-mechanism models provide only an approximate description; similar fitting quality and a dominant Higuchi/diffusion contribution have been reported for oxaliplatin released from other inorganic and composite carriers.^74,75^ The Korsmeyer–Peppas release exponents (*n* < 0.5) for both drugs further support a Fickian diffusion-controlled mechanism. Overall, these findings confirm that drug release from green-synthesized CuO nanoparticles is primarily governed by diffusion-controlled processes with contributions from concentration-dependent desorption, consistent with previously reported nanoparticle-based drug delivery systems.^76,77^.

## Conclusion

This study demonstrates the successful green synthesis of monoclinic CuO nanoparticles using *Avicennia marina* mangrove leaf extract, establishing a sustainable and non-toxic alternative to conventional chemical synthesis routes. XRD analysis confirmed the formation of phase-pure CuO with a monoclinic tenorite structure. SEM analysis revealed quasi-spherical nanoparticles with a size range of 35–87 nm, and EDX confirmed the elemental composition (Cu: 41.86 atomic %). BET analysis yielded a specific surface area of 4.5 m²/g and a total pore volume of 0.018 cm³/g, with a predominant mesoporous architecture that is beneficial for drug loading. The green CuO NPs were successfully loaded with two clinically approved platinum(II) chemotherapeutics. Cisplatin exhibited an entrapment efficiency of 28.3% and a loading capacity of 56 mg/g, while oxaliplatin showed an entrapment efficiency of 17.0% and a loading capacity of 34 mg/g. The higher loading efficiency of cisplatin is consistent with its stronger surface binding affinity at CuO (111) sites. BET analysis confirmed drug incorporation through reductions in surface area: from 4.5 m²/g to 2.45 m²/g (cisplatin) and 3.0 m²/g (oxaliplatin). FTIR analysis confirmed successful drug-nanoparticle coordination through shifts in the N–H, C = O, and Cu–O characteristic bands, while the CuO lattice retained its structural integrity post-loading. In vitro drug release under simulated physiological conditions (pH 7.4 PBS, 37 °C) demonstrated sustained profiles for both drugs: cisplatin reached 70% cumulative release within 20 h, while oxaliplatin achieved approximately 80% over the same period. Kinetic modeling revealed that the release data for cisplatin best fit the first-order model (R² = 0.91) and for oxaliplatin, the Higuchi model gave the closest fit (R² = 0.81), exceeding the first-order fit and indicating that release is predominantly governed by diffusion from the mesoporous CuO matrix. These findings establish *Avicennia marina*-derived CuO nanoparticles as a promising, biocompatible, and sustainable nanocarrier platform for platinum-based chemotherapy, warranting further investigation through in vitro cytotoxicity, haemocompatibility, and in vivo efficacy studies.

## Supplementary Information

Below is the link to the electronic supplementary material.

Supplementary Material 1

## Data Availability

All data supporting the findings of this study are available within the paper and its Supplementary Information.
