# Supplementary material for: Green synthesis of CuO nanoparticles using mangrove leaves for targeted delivery of platinum (II) therapeutics
Source: Sci Rep. 2026 Sep 8;16:28066. doi: 10.1038/s41598-026-67347-7 (PMC13554094; doi:10.1038/s41598-026-67347-7)
Supplement: Supplementary file 1 [file 41598_2026_67347_MOESM1_ESM.docx]

Supplementary Material

Green Synthesis of CuO Nanoparticles Using Mangrove Leaves for Targeted Delivery of Platinum (II) Therapeutics

Shahdan Abdelkareem, Aly Reda, Mayyada M.H. El-Sayed*, Tamer Shoeib*

Department of Chemistry, The American University in Cairo, New Cairo 11835, Egypt.

***Corresponding authors email: [Mayyada@aucegypt.edu](mailto:Mayyada@aucegypt.edu) (M.M.H. El-Sayed), [T.Shoeib@aucegypt.edu](mailto:T.Shoeib@aucegypt.edu) (T.Shoeib)

**SEM Analysis**

Figure S1: SEM of Cisplatin@green-CuO

Figure S2: SEM of Oxaliplatin@green-CuO

**EDX Analysis of Loaded Cisplatin and Oxaliplatin**

Figure S3: EDX Analysis of Cisplatin@green-Cuo

Figure S4: EDX Analysis of Oxaliplatin@green-CuO

## Thermal stability of the loaded nanoparticles

Figure S5: TGA of cisplatin@green CuO NPs and oxaliplatin@green CuO NPs.

**UV-Vis spectra of free oxaliplatin, and oxaliplatin@ green-CuO**, **free cisplatin, and cisplatin@green-CuO**

Figure S6: UV-Vis spectra of a) free oxaliplatin, and oxaliplatin@ green-CuO, b) free cisplatin, and cisplatin@green-CuO

## Drug Release Kinetics

Figure S7: Drug release kinetic model fitting for cisplatin and oxaliplatin loaded on green CuO nanoparticles (A) Zero-order model, (B) First-order model, (C) Higuchi model, and (D) Korsmeyer–Peppas model
